# AllCoPol: inferring allele co-ancestry in polyploids

**DOI:** 10.1186/s12859-020-03750-9

**Published:** 2020-10-07

**Authors:** Ulrich Lautenschlager, Florian Wagner, Christoph Oberprieler

**Affiliations:** Evolutionary and Systematic Botany Group, Institute of Plant Sciences, University of Regensburg, Universitätsstr. 31, 93053 Regensburg, Germany

**Keywords:** Coalescent theory, Gene tree, Multilocus sequence data, Polyploidy, Python, Reticulate evolution, Simulations, Species tree

## Abstract

**Background:**

Inferring phylogenetic relationships of polyploid species and their diploid ancestors (leading to reticulate phylogenies in the case of an allopolyploid origin) based on multi-locus sequence data is complicated by the unknown assignment of alleles found in polyploids to diploid subgenomes. A parsimony-based approach to this problem has been proposed by Oberprieler et al. (Methods Ecol Evol 8:835–849, 2017), however, its implementation is of limited practical value. In addition to previously identified shortcomings, it has been found that in some cases, the obtained results barely satisfy the applied criterion. To be of better use to other researchers, a reimplementation with methodological refinement appears to be indispensable.

**Results:**

We present the AllCoPol package, which provides a heuristic method for assigning alleles from polyploids to diploid subgenomes based on the Minimizing Deep Coalescences (MDC) criterion in multi-locus sequence datasets. An additional consensus approach further allows to assess the confidence of phylogenetic reconstructions. Simulations of tetra- and hexaploids show that under simplifying assumptions such as completely disomic inheritance, the topological errors of reconstructed phylogenies are similar to those of MDC species trees based on the true allele partition.

**Conclusions:**

AllCoPol is a Python package for phylogenetic reconstructions of polyploids offering enhanced functionality as well as improved usability. The included methods are supplied as command line tools without the need for prior programming knowledge.

## Background

Due to the paramount importance of polyploid speciation in plant evolution and the advent of large-scale sequencing technologies (‘next-generation sequencing’) in phylogenetics, there is a growing need for bioinformatics methods that allow the objective reconstruction of reticulate phylogenies caused by allopolyploidy (see [10]). One possible approach suggested by the present workgroup is to explicitly assign sampled alleles of a polyploid taxon to different diploid subgenomes in order to enable species-tree reconstructions or further downstream analyses [8]. It makes use of a parsimony-based principle in species-tree reconstruction (minimizing deep coalescences, MDC, [6, 7]). However, technical shortcomings such as the lack of a complete implementation and its dependence on commercial software hindered the broader application of this method. While its applicability for relatively small data sets has been demonstrated, only poor parsimony scores—sometimes at the level of random solutions—were observed when applied in more challenging cases. This can be explained by its limited optimization strategy, which is ignorant about the interplay of independently calculated partial solutions. For this reason, the AllCoPol package implements a different heuristic approach based on the tabu-search metaheuristic [2] while following the same criterion. The occasional lack of reproducibility caused by the stochasticity of our heuristic further motivated the development of a novel method for calculating consensus results. Other enhancements over the original pipeline include the possibility to address gene tree uncertainty, independence from commercial software, and a more user-friendly interface. AllCoPol as well as the analysis pipeline suggested by Oberprieler et al. [8] uses PhyloNet [13, 15], an actively developed collection of phylogenetic analysis tools, to compute MDC-based species trees. Newer methods implemented in PhyloNet allow to directly infer species networks with a predefined maximum number of reticulations. As proposed by Cao et al. [1], such methods, which can be constrained to consider polyploids as hybridogenic, could be used for the analysis of polyploids. Therefore, AllCoPol is compared with similar analyses using MDC-based species network reconstruction in PhyloNet.

### Implementation

The Python package AllCoPol provides a collection of command line tools. Their usage and a complete list of available options can be displayed using the command line options –help or -h. For application examples along with further guidelines, the reader is referred to the README file.

### Reconstruction of subgenome memberships

The main problem AllCoPol aims to solve is to partition a set of alleles from a *n*-ploid taxon, considering all sampled individuals and loci, into at most *n*/2 subsets representing its hypothetical diploid subgenomes. In doing so, we seek to minimize the number of extra lineages in the resulting MDC species tree. Assuming single-copy loci, for each polyploid individual, at most two alleles per locus may be assigned to the same subgenome. Solutions to the described problem are approximated using the command allcopol, whose options can be specified via command line arguments or within a configuration file. Optimization is done by a simple tabu-search algorithm. Despite its similarity to hillclimbing, the former, when properly parameterized, is able to escape from local optima. Its characteristic feature is a so-called tabu-list, which discourages certain moves in the search space for a specified number of iterations (tabu tenure), aiming to avoid cyclic trajectories.

The analysis requires two input files, one for precomputed gene trees and another one specifying allele names, taxon membership, and ploidy level for each accession (individual) under study. Each taxon may be represented by multiple accessions, each of which may comprise multiple alleles per locus. The calculation of MDC species trees along with their number of extra lineages (i.e., the evaluation of the objective function) is carried out in PhyloNet (in the following, PhyloNet v.3.8.0 is used) using the command Infer_ST_MDC [14, 16], which requires the input gene trees to be rooted. Subgenome memberships are reconstructed for one polyploid taxon at a time. Therefore, alleles belonging to other polyploids in the study are internally pruned from the gene trees. Because, apart from simulation studies, the input gene trees themselves are only estimated, they are better represented by samples from a posterior distribution rather than single point estimates or summary trees [9]. Our script therefore allows multiple gene trees per locus to be considered. The most parsimonious allele partition found during the search, along with its resulting MDC species tree, is returned as output. To fully exploit the advantage of keeping a tabu list, it is recommended to test different values for the tabu-tenure parameter because it is difficult to provide a sensible default. The optimization process stops after a prespecified number of iterations, which represents a trade-off between solution quality (i.e., number of extra lineages) and required runtime. Further recommendations on the optimization parameters can be found in the README file.

### Combination of replicate results

Due to plateaus, local optima, and the inherent stochasticity of our heuristic, the results from repeated runs of allcopol based on the same input data and parameter settings may vary. If they need to be summarized to assess their consistency and to obtain a more robust result, this is complicated by the unknown homology of reconstructed subgenomes across multiple analyses. We therefore implemented a relabeling strategy, which can be applied if multiple reconstructions are based on the same set of loci. Provided that there is a hidden one-to-one correspondence of inferred subgenomes across multiple reconstructions (as expected in case of allopolyploids), proper relabeling allows, for example, the application of common tree consensus methods.

Inferred allele partitions (hard clusterings) can be represented as matrices of binary membership coefficients, where each row represents an allele and each column corresponds to one cluster (subgenome). Assuming that for each column index j, the jth column of each coefficient matrix represents the same subgenome, the membership coefficients can be averaged over all matrices. If these are properly aligned, we expect the distribution of averaged coefficients, which might be viewed as membership probabilities, to be as sharp as possible. In other words, we want to minimize the uncertainty about the cluster membership of alleles in the averaged clustering. For the ith allele, this can be measured by the Shannon entropy H_i_ = − Σ_j_(c_ij_log(c_ij_)), where (c_ij_) are the averaged membership coefficients and c_ij_log(c_ij_): = 0 if c_ij_ = 0. The mean entropy of all considered alleles can be used as objective function, which is minimized by the command align_clusters to match clusters (subgenomes) across multiple reconstructions from allcopol. Its tabular input based on previously inferred allele partitions can be created through the command create_indfile, whose output is also compatible with CLUMPP [4]. Once corresponding subgenomes have been identified, relabel_trees can be used to relabel them in the inferred species trees.

## Results and discussion

### Example analysis

**Fig. 1:**
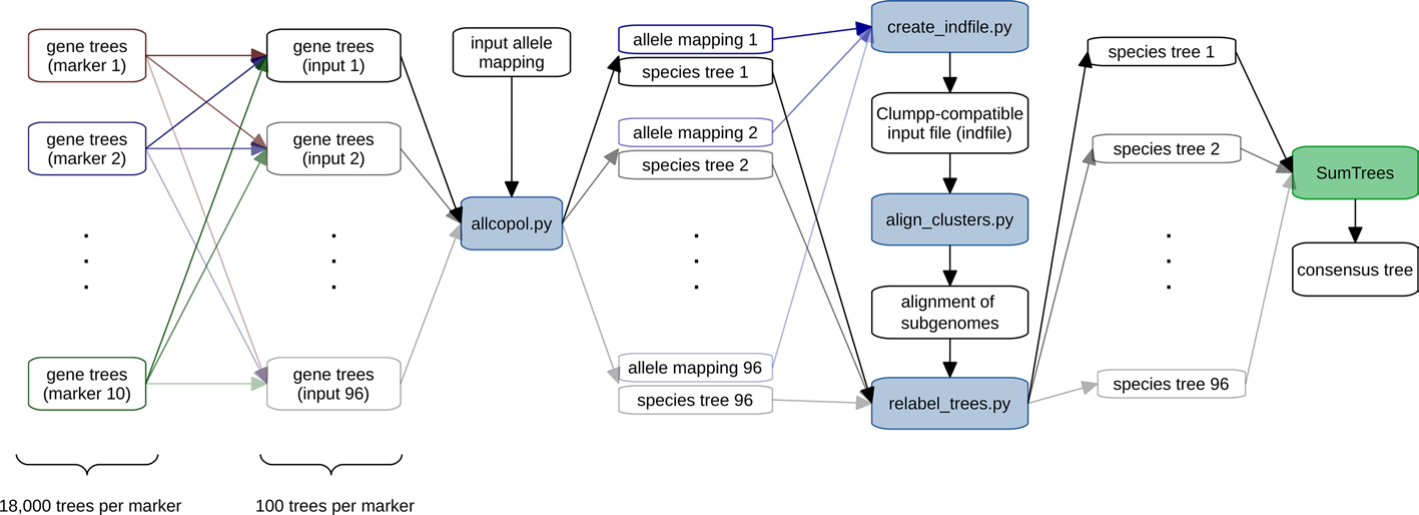
Central tools (color filled boxes) used in the example analysis with their respective input and output files. Blue filled boxes represent scripts which are included in the AllCoPol package

**Fig. 2:**
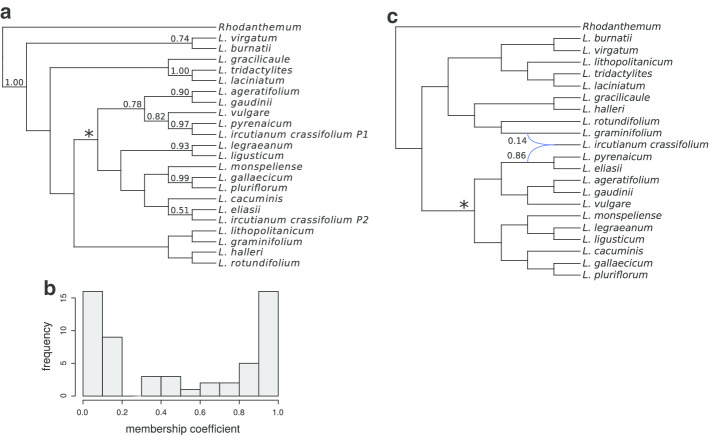
Phylogenetic reconstruction of *L. ircutianum* subsp. *crassifolium*. The presented species tree (**a**) based on optimized allele partitions forms the consensus of 96 individual reconstructions with AllCoPol. Only clade frequencies of at least 0.5 are displayed. The histogram (**b**) illustrates the corresponding frequency distribution of averaged membership coefficients. The presented values refer to the first cluster only, i.e., a value of one denotes that an allele is consistently assigned to the same subgenome labeled as “P1” in the tree above. For comparison, an exemplary network inferred by the PhyloNet command InferNetwork_MP is shown (**c**). Both reticulating branches are annotated with their corresponding inheritance probabilities. The *L. vulgare*-group is marked with asterisk in (**a**) and (**c**)

**Fig. 3:**
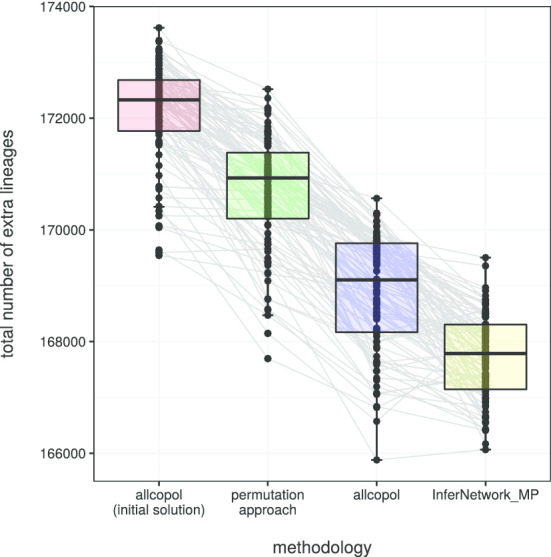
Parsimony of results obtained with different heuristic approaches. All 96 input tree samples were analyzed using the tabu search implemented in AllCoPol, the permutation approach proposed by Oberprieler et al. [8], and the PhyloNet command InferNetwork_MP. For comparison, random solutions, from which AllCoPol starts its optimization, were evaluated. The scores obtained with InferNetwork_MP have been rescaled (multiplication by 100) because, if multiple gene trees per locus are provided, AllCoPol in its current version sums up their number of extra lineages, whereas the former by default uses their average

A way of addressing gene-tree uncertainty is exemplified by an analysis of the tetraploid *Leucanthemum ircutianum* subsp. *crassifolium* (Lange) Vogt together with 21 diploid representatives of the genus and an outgroup. For each of 10 markers, 18,000 gene trees sampled from the posterior were available (Additional files 1 and 2). From these, subsamples of 100 trees per marker were used as input for subgenome and species-tree reconstructions using allcopol. The program also requires accession-specific information, which is specified in Additional file 3. To account for the stochastic nature of both the optimization and the subsampling process, 96 replicate analyses based on different tree subsamples were performed. Thus, we obtained 96 species trees, whose subgenome leaves were then relabeled according to the output of align_clusters. Eventually, a greedy consensus tree was computed with SumTrees from DendroPy v.4.4.0 [12] and visualized with Dendroscope v.3.5.9 [3]. The complete analysis pipeline is illustrated in Fig. 1; optimization parameters were specified according to Additional file 4: Table S1. While one subgenome is consistently inferred as sister of *L. pyrenaicum* Vogt, Konowalik & Oberpr., the phylogenetic placement of the second one appears to be less reliable (Fig. 2a). Nevertheless, as most averaged membership coefficients (Fig. 2b) are close to either one or zero, the obtained allele partitions are quite consistent and argue for an allotetraploid origin of the subspecies. On the other hand, a lack of allelic separation (i.e., averaged membership coefficients around 0.5) would not necessarily imply autopolyploidy and could, for instance, also be caused by insufficient optimization of the MDC criterion, recombination between subgenomes, or other violated assumptions. For each of the 96 input tree samples, the heuristic used by AllCoPol finds a more parsimonious solution than the original permutation approach (Fig. 3), which highlights the importance of the algorithmic re-design for complex input data.

For comparison, the same input data was used to calculate 96 species networks using the PhyloNet command InferNetwork_MP, where we allowed one reticulation and specified *Leucanthemum ircutianum* subsp. *crassifolium* as hybrid species. For each reconstruction, 100 optimization runs were performed (default: 5), otherwise we kept the default settings.

The results differ in several ways from those of AllCoPol. InferNetwork_MP tends to produce more parsimonious solutions (Fig. 3) and, in 95 of 96 of cases, suggests parental lineages from both inside and outside the so-called *L. vulgare*-group (or ‘Group 2’ of [5]), a monophyletic group of closely related diploids. In contrast, AllCoPol places both subgenomes inside the *L. vulgare*-group. An exemplary species network is shown in Fig. 2c, inferred sister clades are summarized in Additional file 5: Tables S2 and S3 and show less reproducibility in case of the network reconstructions. While AllCoPol infers quite balanced allele partitions (Fig. 2b), InferNetwork_MP assigns rather different inheritance probabilities to the parents of a reticulation node, on average 0.83 for the branch inside the *L. vulgare*-group and 0.17 for the other one. The differences mentioned above are probably due to the fact that AllCoPol does not accept solutions where more than two alleles of one locus per individual are assigned to the same subgenome. Without this constraint, reticulation events within closely related taxa are more likely to be dominated by introgression signals from less closely related taxa. It should also be noted that the presented numbers of extra lineages depend on how much computing time is spent on the optimization process. Because InferNetwork_MP is directly implemented in PhyloNet and thus generally faster, its runtime has been less limiting compared to allcopol, where a single analysis consumed about 320 h of CPU time using a Intel Xeon E5-v2650v4 processor. It is recommended to perform analyses of this size on a HPC cluster.

### Validation

**Fig. 4:**
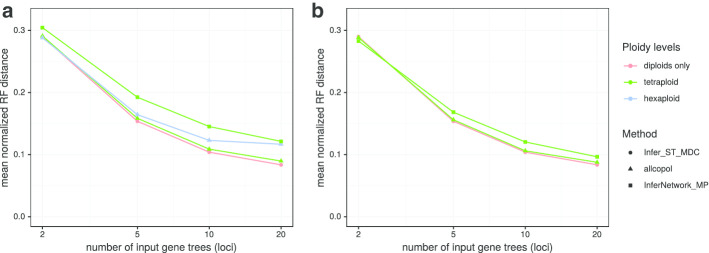
Average topological distance between known and inferred species trees with and without involved polyploids. As all considered trees comprise 8 leaves, the normalized RF distance can only adopt six discrete values (0, 0.2, 0.4, 0.6, 0.8, 1). In contrast to (**a**), polyploids with monophyletic ancestors were excluded in (**b**)

To study the accuracy of phylogenetic reconstructions for tetra- and hexaploids, AllCoPol was applied to a simulated dataset. This was generated using the treesim submodule from DendroPy v.4.4.0 and parameters similar to those used by Than and Nakhleh [14], which implies a considerable amount of incomplete lineage sorting. A pure-birth process with uniform speciation was used to simulate 30 8-taxon species trees, whose edge lengths were scaled to a total depth of 10^6^ generations. Within each species tree, 10,000 coalescent gene trees, each comprising two alleles per taxon, were simulated based on an effective population size of N_e_ = 10^5^. To simulate allopolyploids with completely disomic inheritance (i.e., without recombination among subgenomes), different taxa were treated as subgenomes of a hypothetical polyploid by combining their alleles into one artificial taxon. For each combination of 2 or 3 diploid taxa, allele partitions and species trees were reconstructed using allcopol. To assess the impact of varying numbers of loci, randomly chosen subsets of 2, 5, 10, or 20 gene trees were used as input. For each constellation (species tree, combination of diploids, number of gene trees), 24 reconstructions based on different gene-tree samples were performed. Prior to the analyses, optimization parameters for allcopol were tuned for each ploidy level and sample size (Additional file 6). When different parameter settings resulted in similar average performance, we preferred settings with a higher value of the sample size parameter because, for a fixed number of solution evaluations, this internally leads to less executions of PhyloNet and therefore less runtime spent on redundant calculations. Multiple analyses were executed in parallel on a HPC node with an Intel Xeon E5-v2650v4 processor, where a single reconstruction took between 5 min and about 1 h of CPU time, depending on the ploidy level and the number of gene trees. In addition to the analyses with AllCoPol, MDC species trees for the original diploids were calculated with the PhyloNet command Infer_ST_MDC. The artificial tetraploids were also analysed with the InferNetwork_MP command, where they were specified as hybrid species, allowing one reticulation node in the resulting species network. Each network reconstruction comprised 20 hillclimbing runs. The inferred networks were converted to multi-labelled trees to facilitate the comparison with tree-shaped reference topologies. Reconstruction errors were measured by the normalized Robinson–Foulds (RF) distance [11] between inferred topologies and the known species trees. If polyploids were involved, 2 (tetraploid) or 6 (hexaploid) different mappings between inferred subgenomes and original diploids were possible, from which we used the one that led to the lowest RF distance. The results, illustrated in Fig. 4, show that the missing information about subgenome (taxon) membership in tetra- and hexaploids only moderately increases the errors of reconstructed topologies. In case of the tetraploids, AllCoPol tends to find somewhat more accurate results than InferNetwork_MP. However, this difference decreases if cases are excluded where a polyploid derives from two monophyletic diploid species, which themselves are no longer present in analysis. For such problems, InferNetwork_MP in its applied form cannot infer the correct tree topology, instead, calculating a non-reticulate species tree would be more appropriate. While higher numbers of loci typically lead to lower errors, they not only increase the search space, but also the computing time required to evaluate a single solution.

### Comparison of methods

While AllCoPol outputs a taxon/subgenome tree along with the underlying allele partition, MDC-based network inference in PhyloNet yields a possibly reticulate network without providing allele-specific information. However, explicit allele mappings are a prerequisite to perform certain downstream analyses such as the proposed consensus approach. Varying numbers of reticulations further complicate an empirical comparison of both approaches, especially if ploidy levels beyond tetraploids are concerned. Besides providing a consensus approach for replicate analyses, a distinctive feature of AllCoPol consists in its restriction that a subgenome must not contain more than two alleles at one locus per individual in order to prevent biologically implausible solutions. Despite their conceptual similarity, both methods may infer quite different phylogenies as shown in the example analysis. The presented validation analyses show similar accuracy in either case, but are too simplistic to elucidate under which circumstances one method is preferable over the other and how sensitively they react to violations of their assumptions. To interpret the results obtained, it may be useful to observe how the topologies of the diploid taxa behave, depending on whether polyploids are included.

### Outlook

It should be noted that considering multiple gene trees per locus as well as replicate analyses come at the cost of increased runtime requirements, which may be prohibitive for certain analyses. Future work should therefore pay particular attention to computational efficiency. In addition, the usability could be further improved through a more robust algorithm, which could, for instance, provide an automatic adjustment of optimization parameters.

## Conclusions

AllCoPol is a Python package for phylogenetic reconstructions of polyploids, which improves the methodology of Oberprieler et al. [8] in multiple ways, allowing a simpler and more robust analysis than before. Based on gene trees comprising alleles from either diploid and polyploid taxa, it allows to infer which alleles from a polyploid belong to the same diploid subgenome. Its functionality is provided in the form of command line tools, whose application does not require any programming skills.

### Availability and requirements

Project name: AllCoPol.

Project home page: https://github.com/AGOberprieler/allcopol.

Operating system: Platform independent.

Programming language: Python.

Other requirements: Python 3.5 or higher. To use the main command allcopol, a current version of PhyloNet (3.6.0 or higher) must be obtained. The latter requires a suitable Java installation.

License: MIT. Any restrictions to use by non-academics: none.

## Supplementary information

**Additional file 1:** Empirical data from *Leucanthemum* Mill. (Compositae, Anthemideae). This file describes the data used in the example analysis.

**Additional file 2:** Gene trees used in the example analysis. This file contains the data used in the example analysis comprising 10 markers with 18,000 gene trees each.

**Additional file 3:** Allele mapping used in the example analysis. This file assigns taxon membership, allele identifiers, and ploidy level to each accession under study.

**Additional file 4: Table S1**. Optimization of tuning parameters for the example analysis.

**Additional file 5: Tables S2 and S3**. Inferred sister clades of parental branches of *L. ircutianum* subsp. *crassifolium*.

**Additional file 6: Tables S4–S11**. Optimization of tuning parameters for validation analyses.

## Data Availability

The Python package AllCoPol is freely available at https://github.com/agoberprieler/allcopol. Gene tree populations used in the example analysis are provided in Additional file 2.

## Abbreviations

MDC: Minimizing deep coalescences
HPC Cluster: High-performance computing cluster
RF distance: Robinson–Foulds distance
