# Supplementary material for: AllCoPol: inferring allele co-ancestry in polyploids
Source: BMC Bioinformatics. 2020 Oct 7;21:441. doi: 10.1186/s12859-020-03750-9 (PMC7542712; doi:10.1186/s12859-020-03750-9)
Supplement: Supplementary file 1 — Additional file 1: Empirical data from Leucanthemum Mill. (Compositae, Anthemideae). This file describes the data used in the example analysis. [file 12859_2020_3750_MOESM1_ESM.pdf]

Empirical data from *Leucanthemum* Mill. (Compositae, Anthemideae)

The genus *Leucanthemum* Mill. (´Marguerites´; Compositae, Anthemideae) comprises 42 flowering plant species (Euro+Med, 2006-) distributed all over the European continent and represents an attractive system for studying reticulate evolution on the diploid (Oberprieler et al. 2014, Konowalik et al. 2015, Wagner et al. 2017) and polyploid (Oberprieler et al. 2011, 2014; Greiner et al. 2012, 2013) level. While polyploid chromosome numbers range from tetraploid to docosaploid (22*x*) in the genus, the highest species diversity exists on the tetraploid and hexaploid level.

A total number of 42 accessions of 20 diploid *Leucanthemum* species, along with 22 accessions of nine polyploid taxa were sequenced for nine nuclear markers *(A39, B12, B20, C12, C20, C33, D18, D23, D27)* which were characterised as putative single-copy regions for the sunflower family (Compositae) by Chapman et al. (2007). Additionally, we sequenced three intergenic spacer regions from the plastid genome *(petN-psbM, psbA-trnH, trnQ-rps16).* A majority of sequences for the nuclear single-copy markers in diploids stemmed from the studies of Konowalik et al. (2015) and Wagner et al. (2019) and were sequenced via Roche 454 sequencing. In cases where the 454-sequencing procedure failed to produce suitable reads, these were complemented by traditional Sanger sequencing, often after allele identification via cloning or bioinformatic allele phasing as described in Wagner et al. (2019). Sequence information for tetraploid accessions were also gained either through 454 sequencing (K. Konowalik, unpublished) or through Sanger sequencing of (cloned) PCR products. Gene trees for each nuclear marker and for the concatenated plastid-marker alignment were reconstructed via Bayesian Inference (BI) using BEAST v2.5.2 (Bouckaert et al. 2019). All xml files were generated in BEAUTI v.2.5.2 using a coalescent constant population tree prior, a gamma distribution with shape 2.0 and scale 0.002 for the population size, and otherwise default priors. In all cases, a strict clock model was used. Substitution models were specified according to the Bayesian information criterion (BIC) in JMODELTEST 2.1.10 (Darriba et al. 2012) and two replicate runs for each xml file were performed with 100 million generations and a sample frequency of 10,000. Replicate BEAST runs were finally combined with LOGCOMBINER v.2.5.2 (burn-in of 10%) to generate distributions of 18,000 gene-trees for each marker. The resulting tree populations, on which our analysis of *L. ircutianum* subsp. *crassifolium* (Lange) Vogt is based, are provided in the accompanying zipped folder of allele-trees (Additional file 2). The original gene trees comprised additional taxa, whose alleles have been pruned from the trees. A second file (Additional file 3) assigns taxon membership, allele IDs, and ploidy level to each accession under study.

References

Bouckaert R, Vaughan TG, Barido-Sottani J, Duchêne S, Fourment M, Gavryushkina A, et al. (2019). BEAST 2.5: An advanced software platform for Bayesian evolutionary analysis. – *PLoS computational biology* 15: e1006650.

Darriba D, Taboada GL, Doallo R, Posada D (2012). jModelTest 2: more models, new heuristics and parallel computing. *Nature Methods* 9: 772.

Euro+Med (2006-). Euro+Med PlantBase - the information resource for Euro-Mediterranean plant diversity. Published on the Internet. http://ww2.bgbm.org/EuroPlusMed/ [accessed December 2nd, 2019].

Chapman MA, Chang J, Weisman D, Kesseli RV, Burke JM (2007). Universal markers for comparative mapping and phylogenetic analysis in the Asteraceae (Compositae). – *Theoretical and Applied Genetics* 115: 747–755.

Greiner R, Vogt R, Oberprieler C (2012). Phylogenetic studies in the polyploid complex of the genus *Leucanthemum* Mill. (Compositae, Anthemideae) based on cpDNA sequence variation. – *Plant Systematics and Evolution* 298: 1407-1414.

Greiner R, Vogt R, Oberprieler C (2013). Evolution of the polyploid north-west Iberian *Leucanthemum pluriflorum* clan (Compositae, Anthemideae) based on plastid DNA sequence variation and AFLP fingerprinting. – *Annals of Botany* 111: 1109-1123.

Konowalik K, Wagner F, Tomasello S, Vogt R, Oberprieler C (2015). Detecting reticulate relationships among diploid *Leucanthemum* Mill. (Compositae, Anthemideae) taxa using multilocus species tree reconstruction methods and AFLP fingerprinting. – *Molecular Phylogenetics and Evolution* 92: 308-328.

Oberprieler C, Eder C, Meister J, Vogt R (2011). AFLP fingerprinting suggests the allopolyploid origin of two members of the *Leucanthemum vulgare* aggregate (Compositae, Anthemideae) in Central Europe. – *Nordic Journal of Botany* 29: 370-377.

Oberprieler C, Greiner R, Konowalik K, Vogt R (2014). The reticulate evolutionary history of the polyploid NW Iberian *Leucanthemum pluriflorum* clan (Compositae, Anthemideae) as inferred from nrDNA ETS sequence diversity and eco-climatological niche-modelling. – *Molecular Phylogenetics and Evolution* 70: 478-491.

Wagner F, Härtl S, Vogt R, Oberprieler C (2017). ‚Fix Me Another Marguerite!‘: Species delimitation in a group of intensively hybridising lineages of ox-eye daisies (*Leucanthemum* Mill., Compositae-Anthemideae). – *Molecular Ecology* 26: 4260-4283.

Wagner F, Ott T, Zimmer C, Reichhart V, Vogt R, Oberprieler C (2019). 'At the crossroads towards polyploidy': Genomic divergence and extent of homoploid hybridisation are drivers for the formation of the ox-eye daisy polyploid complex (*Leucanthemum*, Compositae-Anthemideae). – *New Phytologist* 223: 2039-2053.
