# Supplementary material for: AllCoPol: inferring allele co-ancestry in polyploids
Source: BMC Bioinformatics. 2020 Oct 7;21:441. doi: 10.1186/s12859-020-03750-9 (PMC7542712; doi:10.1186/s12859-020-03750-9)
Supplement: Supplementary file 4 — Additional file 4: Table S1. Optimization of tuning parameters for the example analysis. [file 12859_2020_3750_MOESM4_ESM.pdf]

Table S1: Parameter screening for the analysis of *Leucanthemum ircutianum* subsp. *crassifolium* (Lange). In order to reduce the computational burden, only subsamples of 10 trees per marker were used as input to AllCoPol. For each parameter combination, the final number of extra lineages has been averaged over 24 runs, each based on different subsamples from the available gene tree populations. The accepted values are highlighted in bold.

Tuning parameters		Mean number
sample size [%]	tabu tenure	of extra lineages
15	7	17256
**50**	**16**	**17256**
25	7	17257
50	20	17257
15	4	17260
50	10	17260
50	25	17260
25	10	17261
25	4	17262
15	2	17263
25	13	17263
25	2	17263
50	7	17263
15	10	17265
50	2	17265
15	13	17266
25	16	17266
50	4	17266
25	20	17269
50	13	17270
15	16	17273
25	25	17274
15	20	17275
15	25	17278
