# Supplementary material for: AllCoPol: inferring allele co-ancestry in polyploids
Source: BMC Bioinformatics. 2020 Oct 7;21:441. doi: 10.1186/s12859-020-03750-9 (PMC7542712; doi:10.1186/s12859-020-03750-9)
Supplement: Supplementary file 5 — Additional file 5: Tables S2 and S3. Inferred sister clades of parental branches of L. ircutianum subsp. crassifolium. [file 12859_2020_3750_MOESM5_ESM.pdf]

Table S2: Sister clades of the diploid subgenomes of *Leucanthemum ircutianum* subsp. *crassifolium* (Lange) as inferred with AllCoPol. The presented frequencies sum up to 192 (96 analyses, each comprising two subgenomes). Clades belonging to the *Leucanthemum vulgare*-group are marked with an asterisk.

frequency	sister clade
93	L. pyrenaicum*
50	L. eliasii*
33	L. monspeliense*
7	L. gracilicaule, L. halleri
6	L. cacuminis*
1	L. burnatii, L. virgatum
1	L. gracilicaule
1	L. virgatum

Table S3: Sister clades of reticulating parental branches of *Leucanthemum ircutianum* subsp. *crassifolium* (Lange) as inferred by the PhyloNet command InferNetwork_MP. The presented frequencies sum up to 192 (96 analyses, each comprising one binary reticulation). Clades belonging to the *Leucanthemum vulgare*-group are marked with an asterisk.

frequency	sister clade
38	L. eliasii, L. pyrenaicum*
37	L. graminifolium
32	L. pyrenaicum*
29	L. lithopolitanicum
18	L. cacuminis, L. eliasii*
11	L. virgatum
9	L. gracilicaule
9	L. halleri
6	L. eliasii*
2	L. ageratifolium*
1	L. vulgare*
