# Supplementary material for: AllCoPol: inferring allele co-ancestry in polyploids
Source: BMC Bioinformatics. 2020 Oct 7;21:441. doi: 10.1186/s12859-020-03750-9 (PMC7542712; doi:10.1186/s12859-020-03750-9)
Supplement: Supplementary file 6 — Additional file 6: Tables S4–S11. Optimization of tuning parameters for validation analyses. [file 12859_2020_3750_MOESM6_ESM.pdf]

Table S4: Parameter screening for the analysis of simulated tetraploids based on 2 input gene trees. For four exemplary inputs, the final number of extra lineages, averaged over 24 replicate runs, is shown. Each species tree reconstruction comprised 1200 evaluated candidate solutions. The accepted parameter combination and its performance are highlighted in bold.

Tuning parameters		Average number of extra lineages				
sample size	tabu tenure	scenario 1	scenario 2	scenario 3	scenario 4	mean
1	1	14.00	25.00	13.00	10.00	15.50
1	2	14.00	25.00	13.00	10.00	15.50
1	3	14.00	25.00	13.00	10.00	15.50
2	1	14.00	25.00	13.00	10.00	15.50
2	2	14.00	25.00	13.00	10.00	15.50
2	3	14.00	25.00	13.00	10.00	15.50
4	1	14.00	25.00	13.00	10.00	15.50
4	2	14.00	25.00	13.00	10.00	15.50
4	3	14.00	25.00	13.00	10.00	15.50
6	1	14.00	25.00	13.00	10.00	15.50
6	2	14.00	25.00	13.00	10.00	15.50
6	3	14.00	25.00	13.00	10.00	15.50
8	1	14.00	25.00	13.00	10.00	15.50
**8**	**2**	**14.00**	**25.00**	**13.00**	**10.00**	**15.50**
8	3	14.00	25.00	13.00	10.00	15.50
8	4	14.00	25.00	13.00	10.00	15.50
6	4	14.00	25.00	13.38	10.12	15.62
2	4	14.29	25.21	13.79	10.38	15.92
4	4	14.29	25.17	13.75	10.50	15.93
1	4	14.46	25.17	15.42	11.00	16.51

Table S5: Parameter screening for the analysis of simulated tetraploids based on 5 input gene trees. For four exemplary inputs, the final number of extra lineages, averaged over 24 replicate runs, is shown. Each species tree reconstruction comprised 3000 evaluated candidate solutions. The accepted parameter combination and its performance are highlighted in bold.

Tuning parameters		Average number of extra lineages				
sample size	tabu tenure	scenario 1	scenario 2	scenario 3	scenario 4	mean
5	2	49.00	52.00	41.00	42.00	46.00
10	2	49.00	52.00	41.00	42.00	46.00
10	4	49.00	52.00	41.00	42.00	46.00
10	6	49.00	52.00	41.00	42.00	46.00
10	8	49.00	52.00	41.00	42.00	46.00
15	2	49.00	52.00	41.00	42.00	46.00
15	4	49.00	52.00	41.00	42.00	46.00
15	6	49.00	52.00	41.00	42.00	46.00
20	4	49.00	52.00	41.00	42.00	46.00
**20**	**6**	**49.00**	**52.00**	**41.00**	**42.00**	**46.00**
3	2	49.00	52.00	41.21	42.00	46.05
15	8	49.00	52.00	41.00	42.21	46.05
20	8	49.00	52.00	41.00	42.21	46.05
5	6	49.08	52.00	41.00	42.17	46.06
5	4	49.00	52.00	41.21	42.17	46.09
20	10	49.00	52.00	41.00	42.75	46.19
20	2	49.00	52.00	41.92	42.62	46.39
5	8	49.17	52.00	41.42	43.00	46.40
3	4	49.17	52.00	41.83	43.08	46.52
3	6	49.25	52.00	42.25	43.00	46.62
15	10	49.25	52.00	42.29	43.83	46.84
3	8	49.42	52.29	42.88	43.21	46.95
10	10	50.08	52.50	44.25	45.88	48.18
5	10	55.67	55.38	49.96	48.79	52.45
3	10	56.79	55.17	48.71	49.79	52.61

Table S6: Parameter screening for the analysis of simulated tetraploids based on 10 input gene trees. For four exemplary inputs, the final number of extra lineages, averaged over 24 replicate runs, is shown. Each species tree reconstruction comprised 6000 evaluated candidate solutions. The accepted parameter combination and its performance are highlighted in bold.

Tuning parameters		Average number of extra lineages				
sample size	tabu tenure	scenario 1	scenario 2	scenario 3	scenario 4	mean
30	10	97.33	111.38	90.00	83.00	95.43
30	15	97.54	111.17	90.00	83.00	95.43
20	15	97.58	111.17	90.00	83.00	95.44
10	2	97.08	111.83	90.00	83.00	95.48
**40**	**10**	**97.75**	**111.42**	**90.00**	**83.00**	**95.54**
40	15	98.08	111.00	90.00	83.21	95.57
20	10	97.17	111.71	90.00	83.50	95.59
30	5	97.83	112.33	90.00	83.00	95.79
20	5	97.67	112.54	90.00	83.00	95.80
20	2	98.08	112.50	90.00	83.00	95.90
10	5	97.25	112.04	91.00	83.46	95.94
30	2	99.00	112.33	90.00	83.00	96.08
5	2	97.46	111.88	91.62	83.58	96.14
40	5	98.42	112.67	90.00	84.04	96.28
40	20	99.08	113.83	90.50	83.96	96.84
10	10	98.96	113.00	91.83	85.17	97.24
40	2	99.08	114.54	90.00	85.67	97.32
30	20	99.50	113.75	91.54	85.04	97.46
10	15	99.33	113.33	92.17	85.88	97.68
5	5	99.46	113.04	95.54	87.75	98.95
20	20	101.12	114.33	95.00	86.83	99.32
5	10	101.46	115.54	98.83	96.08	102.98
5	15	102.50	116.12	103.33	99.46	105.35
10	20	104.08	117.29	103.62	100.08	106.27
5	20	107.17	120.00	118.83	114.58	115.15

Table S7: Parameter screening for the analysis of simulated tetraploids based on 20 input gene trees. For four exemplary inputs, the final number of extra lineages, averaged over 24 replicate runs, is shown. Each species tree reconstruction comprised 12000 evaluated candidate solutions. The accepted parameter combination and its performance are highlighted in bold.

Tuning parameters		Average number of extra lineages				
sample size	tabu tenure	scenario 1	scenario 2	scenario 3	scenario 4	mean
10	2	169.25	215.58	192.33	181.46	189.66
60	15	170.46	216.96	192.75	182.17	190.58
20	2	169.29	218.42	192.00	182.79	190.63
20	5	169.67	218.08	193.08	181.92	190.69
**80**	**20**	**171.67**	**216.96**	**192.71**	**181.58**	**190.73**
80	15	171.92	216.71	192.29	182.38	190.82
40	5	171.17	217.29	192.00	183.62	191.02
60	10	171.58	217.25	192.42	183.04	191.07
40	10	170.33	217.42	193.67	183.04	191.11
80	10	172.25	217.88	192.21	182.50	191.21
60	20	171.54	216.62	194.75	182.00	191.23
40	15	170.92	215.92	194.67	183.71	191.30
40	20	170.42	217.42	195.29	182.92	191.51
60	5	176.42	217.33	192.00	182.33	192.02
10	5	171.29	216.71	197.25	184.25	192.38
20	10	171.46	216.17	198.17	185.25	192.76
40	2	175.79	219.67	192.00	183.96	192.85
80	5	178.25	218.08	195.67	182.42	193.60
60	2	176.92	218.08	196.92	183.58	193.88
80	2	176.38	218.88	198.25	182.71	194.05
20	15	172.17	217.46	200.58	187.12	194.33
20	20	174.58	219.54	206.00	187.46	196.90
10	10	176.08	220.62	204.62	188.71	197.51
10	15	178.83	224.08	214.71	193.17	202.70
10	20	183.12	226.12	214.08	194.21	204.39

Table S8: Parameter screening for the analysis of simulated hexaploids based on 2 input gene trees. For four exemplary inputs, the final number of extra lineages, averaged over 24 replicate runs, is shown. Each species tree reconstruction comprised 3600 evaluated candidate solutions. The accepted parameter combination and its performance are highlighted in bold.

Tuning parameters		Average number of extra lineages				
sample size	tabu tenure	scenario 1	scenario 2	scenario 3	scenario 4	mean
3	2	14.00	24.00	13.00	10.00	15.25
3	4	14.00	24.00	13.00	10.00	15.25
3	6	14.00	24.00	13.00	10.00	15.25
6	2	14.00	24.00	13.00	10.00	15.25
6	4	14.00	24.00	13.00	10.00	15.25
6	6	14.00	24.00	13.00	10.00	15.25
12	2	14.00	24.00	13.00	10.00	15.25
12	4	14.00	24.00	13.00	10.00	15.25
12	6	14.00	24.00	13.00	10.00	15.25
18	2	14.00	24.00	13.00	10.00	15.25
18	4	14.00	24.00	13.00	10.00	15.25
18	6	14.00	24.00	13.00	10.00	15.25
24	2	14.00	24.00	13.00	10.00	15.25
24	4	14.00	24.00	13.00	10.00	15.25
**24**	**6**	**14.00**	**24.00**	**13.00**	**10.00**	**15.25**
24	8	14.00	24.00	13.00	10.00	15.25
18	8	14.08	24.00	13.00	10.00	15.27
12	8	14.08	24.00	13.00	10.08	15.29
18	10	14.17	24.00	13.25	10.00	15.35
6	8	14.00	24.17	13.38	10.08	15.41
24	10	14.42	24.33	13.00	10.00	15.44
3	8	14.00	24.25	14.17	10.00	15.60
12	10	14.54	25.00	13.62	10.21	15.84
6	10	14.54	25.92	15.38	10.79	16.66
3	10	15.00	26.96	17.62	11.75	17.83

Table S9: Parameter screening for the analysis of simulated hexaploids based on 5 input gene trees. For four exemplary inputs, the final number of extra lineages, averaged over 24 replicate runs, is shown. Each species tree reconstruction comprised 9000 evaluated candidate solutions. The accepted parameter combination and its performance are highlighted in bold.

Tuning parameters		Average number of extra lineages				
sample size	tabu tenure	scenario 1	scenario 2	scenario 3	scenario 4	mean
8	2	44.04	49.00	38.12	41.00	43.04
15	5	44.21	49.00	38.17	41.17	43.14
**60**	**15**	**44.08**	**49.00**	**38.58**	**41.08**	**43.19**
45	10	44.58	49.00	38.17	41.00	43.19
45	15	44.46	49.17	38.25	41.00	43.22
30	10	44.58	49.08	38.25	41.00	43.23
15	2	44.83	49.00	38.00	41.42	43.31
60	10	45.17	49.00	38.00	41.17	43.33
30	5	45.00	49.00	38.00	41.42	43.35
60	20	45.04	49.25	38.25	41.42	43.49
30	15	44.29	49.50	39.21	41.00	43.50
45	5	45.50	49.00	38.54	41.25	43.57
15	10	44.67	49.96	39.25	41.04	43.73
45	2	45.58	49.25	38.58	41.96	43.84
45	20	45.12	49.46	39.38	41.42	43.84
30	2	46.42	49.00	38.58	41.67	43.92
8	5	44.46	50.17	40.17	41.04	43.96
60	30	46.00	49.42	39.46	42.00	44.22
60	2	46.08	49.67	39.17	42.08	44.25
60	5	47.29	49.00	39.17	41.71	44.29
45	30	46.62	49.33	39.58	41.75	44.32
15	15	45.00	51.29	41.25	41.38	44.73
30	20	46.38	50.21	41.58	41.42	44.90
30	30	46.04	50.75	40.92	42.38	45.02
8	10	45.29	52.17	42.75	41.92	45.53
15	20	47.04	53.00	43.96	43.29	46.82
8	15	46.88	54.21	45.08	43.46	47.41
15	30	48.12	54.00	46.62	43.38	48.03
8	20	49.38	58.96	48.71	45.75	50.70
8	30	52.29	61.29	50.96	47.50	53.01

Table S10: Parameter screening for the analysis of simulated hexaploids based on 10 input gene trees. For four exemplary inputs, the final number of extra lineages, averaged over 24 replicate runs, is shown. Each species tree reconstruction comprised 18000 evaluated candidate solutions. The accepted parameter combination and its performance are highlighted in bold.

Tuning parameters		Average number of extra lineages				
sample size	tabu tenure	scenario 1	scenario 2	scenario 3	scenario 4	mean
60	20	94.29	115.83	92.96	83.00	96.52
15	2	94.21	113.33	93.71	85.42	96.67
15	5	94.33	115.00	94.38	83.08	96.70
**90**	**20**	**93.83**	**116.25**	**94.29**	**83.21**	**96.90**
30	10	94.29	115.00	93.92	85.08	97.07
30	15	94.25	118.04	95.12	83.00	97.60
60	15	93.92	115.21	93.54	88.04	97.68
90	15	94.08	114.71	94.29	88.25	97.83
60	10	94.21	115.29	94.54	87.29	97.83
30	5	94.33	114.92	95.08	87.38	97.93
120	20	94.21	118.29	94.08	85.12	97.93
120	15	94.25	116.50	95.67	85.46	97.97
120	10	94.38	115.92	96.46	87.08	98.46
90	10	93.92	117.92	95.21	86.79	98.46
15	10	94.54	119.79	97.50	83.46	98.82
60	5	94.12	120.17	94.42	88.17	99.22
30	2	94.62	118.54	94.92	88.83	99.23
120	30	95.08	120.88	95.92	85.50	99.34
30	20	94.54	122.17	97.46	83.71	99.47
60	2	94.42	121.88	94.92	89.08	100.07
90	5	94.62	121.62	97.29	88.54	100.52
60	30	95.29	121.88	99.25	85.75	100.54
90	2	94.62	123.75	98.29	87.75	101.10
120	5	94.92	123.04	99.83	87.21	101.25
90	30	94.25	125.71	99.33	85.75	101.26
15	15	94.75	125.50	102.00	84.83	101.77
120	2	94.96	126.75	98.58	90.17	102.61
15	20	96.25	129.83	106.33	87.71	105.03
30	30	96.21	132.00	105.79	90.17	106.04
15	30	98.58	140.38	114.38	96.17	112.38

Table S11: Parameter screening for the analysis of simulated hexaploids based on 20 input gene trees. For four exemplary inputs, the final number of extra lineages, averaged over 24 replicate runs, is shown. Each species tree reconstruction comprised 36000 evaluated candidate solutions. The accepted parameter combination and its performance are highlighted in bold.

Tuning parameters		Average number of extra lineages				
sample size	tabu tenure	scenario 1	scenario 2	scenario 3	scenario 4	mean
30	5	167.67	228.92	191.46	179.04	191.77
30	10	167.71	229.83	194.71	177.29	192.39
30	2	167.54	231.08	190.92	180.50	192.51
**120**	**15**	**167.88**	**232.00**	**192.71**	**178.71**	**192.82**
60	15	167.25	232.42	193.46	179.12	193.06
60	10	169.42	232.25	192.25	178.62	193.14
60	5	169.17	229.62	194.92	178.92	193.16
60	20	167.21	233.88	197.00	177.17	193.81
30	15	168.00	232.58	197.46	177.33	193.84
120	20	168.04	232.08	196.79	179.08	194.00
240	20	169.46	237.00	193.08	178.08	194.41
120	10	168.92	236.42	194.17	178.42	194.48
180	15	169.96	236.42	194.00	178.33	194.68
180	30	166.12	237.92	198.67	176.92	194.91
180	20	168.50	239.54	195.21	179.17	195.60
180	10	168.67	235.62	200.29	177.96	195.64
240	15	168.54	239.08	197.08	178.50	195.80
120	30	167.04	238.79	200.96	177.38	196.04
240	10	168.29	237.67	199.50	179.00	196.11
60	2	169.29	238.08	198.33	179.71	196.35
180	5	167.79	237.38	202.71	178.62	196.62
240	30	167.42	240.83	200.50	177.92	196.67
120	5	169.25	240.17	199.88	178.17	196.86
30	20	169.12	240.04	200.75	178.08	197.00
240	5	168.25	240.83	200.46	179.33	197.22
60	30	166.92	240.79	204.58	177.33	197.41
180	2	166.83	245.33	204.71	178.71	198.90
120	2	169.08	244.58	205.12	178.88	199.42
240	2	169.08	247.21	211.04	178.42	201.44
30	30	169.67	246.46	212.08	179.46	201.92
